# Combining Copper and Zinc into a Biosensor for Anti-Chemoresistance and Achieving Osteosarcoma Therapeutic Efficacy

**DOI:** 10.3390/molecules28072920

**Published:** 2023-03-24

**Authors:** Yan Yik Lim, Ahmad Mujahid Ahmad Zaidi, Azizi Miskon

**Affiliations:** 1 Faculty of Defence Science and Technology, National Defence University of Malaysia, Sungai Besi Camp, Kuala Lumpur 57000, Malaysia; 2 Faculty of Engineering, National Defence University of Malaysia, Sungai Besi Camp, Kuala Lumpur 57000, Malaysia

**Keywords:** metal-based drugs, CuZn, anti-chemoresistance, Osteosarcoma Therapy, ligand biosensors

## Abstract

Due to its built-up chemoresistance after prolonged usage, the demand for replacing platinum in metal-based drugs (MBD) is rising. The first MBD approved by the FDA for cancer therapy was cisplatin in 1978. Even after nearly four and a half decades of trials, there has been no significant improvement in osteosarcoma (OS) therapy. In fact, many MBD have been developed, but the chemoresistance problem raised by platinum remains unresolved. This motivates us to elucidate the possibilities of the copper and zinc (CuZn) combination to replace platinum in MBD. Thus, the anti-chemoresistance properties of CuZn and their physiological functions for OS therapy are highlighted. Herein, we summarise their chelators, main organic solvents, and ligand functions in their structures that are involved in anti-chemoresistance properties. Through this review, it is rational to discuss their ligands’ roles as biosensors in drug delivery systems. Hereafter, an in-depth understanding of their redox and photoactive function relationships is provided. The disadvantage is that the other functions of biosensors cannot be elaborated on here. As a result, this review is being developed, which is expected to intensify OS drugs with higher cure rates. Nonetheless, this advancement intends to solve the major chemoresistance obstacle towards clinical efficacy.

## 1. Introduction

Currently, the importance of metal-based drugs (MBD) in medical applications and commercial markets is increased by the advancement of nanotechnology [1,2]. The antiquity MBD, which is composed of elements such as iron, lithium, vanadium, gold, magnesium, and bismuth, has long been used to treat ailments such as anaemia, bipolar disorder, diabetes, rheumatoid arthritis, stroke, and ulcers, respectively [3]. Among them, the most well-known are platinum-based drugs (PBD) such as cisplatin, carboplatin, and oxaliplatin, which are the most commonly used to treat cancer [4]. PBD in general or cisplatin in particular was approved in 1978, and it is the most preferable drug candidate [5] for a wide range of human diseases in chemotherapeutic applications [6,7]. As a result, this triumph has had a large impact on cancer treatment regimens [8] and influenced the discovery of a new MBD [9]. In this perspective, the current clinical trials are limited to several putative compounds and mechanisms of action in the development of cancer drugs [10] and diagnostic agents [1]. This is not a good sign for drug development [11]. Alternative potencies, such as copper and zinc (CuZn) compounds, should be explored to bring new action mechanisms and chemotherapeutic approaches [12,13]. Without doubt, copper-based drugs (CBD) [14] and zinc-based drugs (ZBD) [15] have active metabolic and physiological functions to develop into the most promising pharmacological non-steroidal anti-inflammatory drugs (NSAID) [12,16]. In addition, zinc stimulates bone formation and mineralization and improves osteoblast differentiation [17]. Combining copper with zinc will avoid genetic disorders and release oncogenic enzymes to regulate and restore homeostasis [18,19]. A better understanding of their combination and how they play important roles in physiological functions will enhance OS drug development [20]. This will alter MBD’s perspectives and generate new drug discovery insight maps [21]. In modern medicine, the understanding of metal-ion functions [22] and diagnosis at the molecular level [23] have become inevitable consequences of delivering new MBD in medicinal bioinorganic chemistry [24,25]. There is still inadequate effort devoted at mechanistic levels [26] towards providing an alternative, targeted, and rational approach [27] to supplement screening of novel chemical entities for biological activity [21].

Chemoresistance in OS immunotherapy [28] is the main problem in MBD in general and PBD in particular [29]. This problem increases after long-term treatments due to its acquired and accumulated nature [30,31]. Chemoresistance develops over time, limiting clinical application and raising concerns about efficacy and systemic toxicity [32,33]. Many attempts intend to solve this problem, but none combine CuZn into a biosensor to stimulate drug release for OS therapy (OST) [30,34]. In our previous paper, we presented some evidence of combinational and targeted biosensors to trigger and stimulate drug release [35]. Our efforts to develop a multifunctional biosensor for OST, however, will be insufficient unless we investigate the physiological functions of CuZn [36]. On the contrary, not much research on CuZn has successfully provided details of multifunctional biosensors for balancing and controlling drug release during cancer invasion [33]. Despite this, their chelation structures [37], aromatic organic solvents [38], and donor atoms of ligands [18] remain unclear, making structural strength [39] the primary barrier to therapeutic efficacy. Thus, more recent approaches are needed to elucidate them and further intensify their degradation factors and functions [40,41].

In this review, the therapeutic efficacy and anti-chemoresistance of OS are discussed but not the OS pathology [35]. It is prudent to discuss the physiological functions of copper and zinc elements in OST but not their general chemotherapeutic potencies. Notably, there are too many papers discussing them; therefore, this paper will reconstruct their combination to elaborate on anti-chemoresistance and OST precisely. First of all, their ions serve as chelators for their structures, such as chelating and metal–organic frameworks (MOF), which influence their anti-chemoresistance. Secondly, the structures of the main organic solvents, such as planar aromatic, Schiff-based, and Schiff-paired, also influence their anti-chemoresistance. Thirdly, their ligand degradation factors are discussed individually to enrich our understanding. The basic functions of their ligands are expected to serve as biosensors, which are clearly elucidated through this review. That is to say, the key biosensor functions, such as redox and photo, serve as guidance for the next-generation OST biosensors [42]. In fact, it is important to design an enzymatic stimulation biosensor for OST. The reality is that this and other functions of biosensors could not be elaborated due to space limitations. As a result, those interested can find more information in our papers [35,36]. This is the rationale for developing a biosensor with sustained efficacy and minimal adverse effects. There are some remaining unclear problems resulting in a major obstacle towards clinical translation, which will be discussed later.

## 2. Physiological Functions of Copper and Zinc Elements

Copper and zinc both regulate each other’s levels in our bodies’ metabolisms [43]. Chronic high zinc consumption is toxic, as is myeloneuropathy [44], and inhibits copper absorption, causing copper deficiency or hypocupraemia [45,46]. Additional zinc was added to oral D-penicillamine [47] in Wilson disease therapy [48], which found efficacy in decreasing unnecessary copper absorption and chelation, resulting in side effects [49]. As a result, copper deficiency and excess are negatively related to zinc excess and deficiency. Consequently, CuZn is used in therapies in our bodies, and their toxicity should be minimised and their use regulated to improve efficacy [13].

Copper is the third most abundant metal-tracing element in our bodies [50]. It is an indispensable microelement for the development and replication of all eukaryotes [51]. It is also required for the growth of biological functions and energy generation in the mitochondrial respiratory chain [52]. Its efficiency uptakes and transports zinc that is bound to chaperone proteins to regulate homeostasis and avoid genetic disorders [19,53]. The copper oxidative states of Cu^1+^ and Cu^2+^ are critical catalytic cofactors for enzyme functions in the chemistry of redox proteins [54]. Copper is present in our bodies in an average amount of 100 mg [55]. A copper deficiency stops cell proliferation and spreading, but exceeding cellular needs will damage cell membranes, proteins, and nucleic acids [49]. This excessive copper will induce cyclins and cyclin-dependent kinase (CDK)-2 in intracellular cells [56]. Thus, copper deficiency and excess cause the copper-transporting P-type ATPase (ATP)-7A and ATP7B gene mutations, resulting in Menkes’ and Wilson’s diseases, respectively [57].

Zinc is the second most abundant and indispensable metal-tracing element after iron in our bodies [58,59]. It is found in thousands of proteins and enzymes, including 85% of muscle and bone, 11% of skin and liver, and residue in other tissues [60]. It participates in their structure, catalysis, and intracellular regulation of lymphocyte apoptosis [54]. Moreover, it plays a significant role in growth and various biological functions of the immune system [17]. It also plays the roles of immune mediator and neuromodulator in the immune system, integrating enzymes, thymic peptides, cytokines, and neurons [61]. As a result, a zinc deficiency causes immune cell suppression, cellular growth retardation, and homeostasis disruption, all of which contribute to the development of diseases and cancer [53]. In contrast, excessive Zn^2+^ inhibits electron delivery to uncoupled mitochondria and suppresses cytocompatibility [62]. Meanwhile, Zn^2+^ has biphasic effects on cell proliferation, adhesion, and viability [63,64].

## 3. Copper and Zinc for Anti-Chemoresistance in Osteosarcoma Therapies

MBD is traditionally referred to as a PBD, which is the most commonly used therapy in the treatment of hard tumours [29]. Cisplatin, oxaliplatin, and carboplatin are the commercially available PBDs, which are effective chemotherapy approaches for anti-cancer drugs [4]. However, their use is discouraged by their intrinsic and acquired chemoresistance [5]. CuZn is used as a chelating agent in cellular trafficking to overcome PBD chemoresistance [65]. Even though CuZn can overcome this chemoresistance, the copper level is critical in our bodies and must be carefully regulated [66]. The problem of copper levels must be solved before producing MBD made of CuZn [67]. However, both copper and zinc are important metal-tracing elements and should not be neglected in cancer therapies. Further studies on them should be widely conducted to replace the more toxic PBD.

CBD is popularly used for anti-cancer [55] due to its anti-chemoresistance, redox, and biocompatibility properties [68,69]. For instance, the common oral administrations used for Wilson’s disease are d-penicillamine, tetrathiomolybdate, and triethylene tetramine [70]. In this therapy, the copper chelator binds the excess copper to maintain genetic homeostasis [71]. Due to the urine and biliary excretions, the outcomes of this therapy are low toxicity, fewer side effects, and easy diagnosis [70,72]. As a result, this chelator modulates homeostasis by regulating the expression of high-affinity copper uptake protein (CTR)-1 [73]. Thus, this causes the cisplatin chemoresistance to be removed by the invasive tumours that actively consume copper delivery in ATPase7A and ATPase7B to release the oncogenic enzymes and increase therapeutic efficacy [74]. As a result, the activity and trafficking of the ATP7A and ATP7B expressions are primarily used to assess the efficacy of PBDs [75]. For instance, the gene miR-148a-3p is used to inhibit ATP7A expression and increase therapeutic efficacy [76]. For ATP7B expression, Tranilast, Tremisaltan, and Amphotericin B are used to inhibit and increase therapeutic efficacy by inducing DNA damage [77,78]. Furthermore, increasing CTR1 expression and cytosolic Cu chaperone antioxidant protein 1 (ATOX1) levels reduced cisplatin chemoresistance [74,79]. Thus, the regulations of ATP7A, ATP7B, CTR1, and ATOX1 are vital and involved in the chain of cisplatin transportation [73]. 

ZBD is commonly used for immunological effects, which prevent disease infections in cancer treatments [80]. Its advantages are low toxicity generally, fewer side effects, and a lack of redox activity [81]. This unique chemical feature of being redox-inactive creates an antioxidant protection system [82]. This divalent zinc ion Zn^2+^ has an electron affinity that is similar to but not identical to that of the copper ion Cu^2+^, which eliminates the possibility of free radical reactions [83,84]. Despite the wide range of ZBD therapies, OSTs are highlighted [64]. In particular, zinc maintains normal endothelial integrity by using basic fibroblast growth factor to stimulate endothelial cell proliferation [64]. Many studies show zinc can stimulate bone formation and mineralization, interact with vial hormones for bone growth, and improve osteoblast differentiation [63]. Zinc also promotes the genes for bone markers [85] such as alkaline phosphatase, collagen type I, osteocalcin, and osteopontin [63]. In comparison, the zinc cation is unique because it has an apparent inhibitory effect on osteoclastic bone resorption at a concentration as low as 10–14 M [86,87]. In conclusion, CBD and ZBD have excellent anti-chemoresistance in OST and great potential to replace cisplatin.

## 4. Copper, Zinc, and CuZn Structures in Anti-Chemoresistance

**Figure 1:**
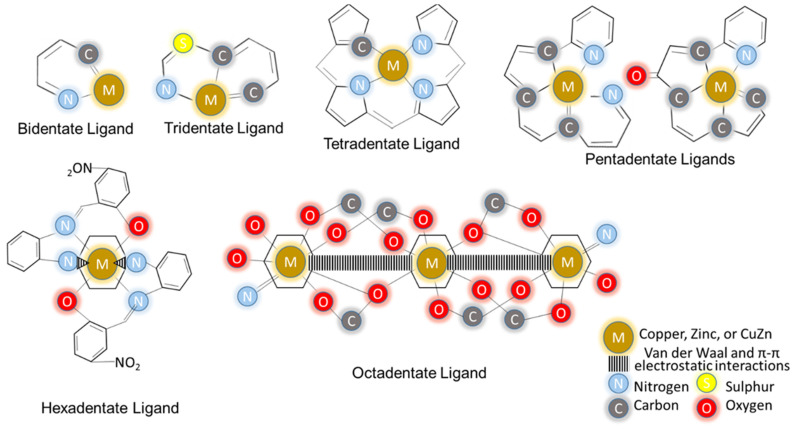
Copper, zinc, and CuZn ions as metal chelators bind with aromatic rings at C, N, O, and S donor atoms with bi-, tri-, tetra-, penta- [95], hexa- [96], and octa-dentate ligands [97]. Reprinted with permission.

Recent efforts have been made to modify the chelating and MOF structures of CuZn in order to overcome chemoresistance [88,89]. These modifications aim to restore the main mechanisms of trigger signals that induce the organic compound reactions in cell apoptosis [89,90]. As previously stated, copper and zinc are necessary for metabolic and immune functions, respectively [43,81]. Both their excess and deficiency harm our bodies [51,56]. Thus, the chelation and MOF approaches can also be used for balancing and controlling their dosage release during cancer invasion [32,91]. In fact, these approaches use different chelators and MOFs, which are supported by the bulk of the evidence [92,93]. Both copper and zinc use the appropriate chelators and MOFs to remove their excess and ionophore compounds [92] to increase their concentration [93]. As a result, chelation and MOF therapies with donor atoms have emerged as the primary cancer therapy strategies in tumoral pathologies [94]. An illustration was drawn to elucidate metal chelators binding with aromatic rings at C, N, O, and S donor atoms with bi-, tri-, tetra-, penta- [95], hexa- [96], and octa-dentate ligands [97], as shown in Figure 1.

### 4.1. Copper and Zinc in Chelating Structures

Copper chelating structures have cuprous Cu^1+^ (copper(I)) and cupric Cu^2+^ (copper(II)) that mainly bond with C, N, O, or S donor atoms [98,99]. This is because of the Jahn–Teller effect in their d-orbitals; copper ions exist in two coordination redox states [22]. Their ligands are cysteine and methionine for S donor atoms [100] and histidine, glutamic acid, and aspartic acid for N or O donor atoms [101]. Due to their copper(I) and copper(II) redox states, they have a higher IC_50_ value and inhibitory activity, resulting in greater potency, clinical effectiveness, and less toxicity than other anti-proliferative drugs [53,102]. These different oxidation states increase thermal stability and ease the formation of CBDs during catalytic processes, resulting in their widespread use [103,104,105]. However, the chelating mechanisms of copper(I) and copper(II) are complex and intertwined [102]. For instance, the 6-transmembrane epithelial antigen of prostate reductase (STEAP) converts copper into copper(I) in serum [25]. In the tissue cell copper uptake mechanism, CTR1 transports copper(I) but not copper(II) [106]. For the same mechanism, CTR1 only works with copper(II) in conjunction with a metalloreductase [107]. As a result, both the copper uptake mechanisms of transporter and reductase can regulate intracellular copper levels in cancer cells [108]. Despite the fact that CTR1, ATOX1, ATP7A, and ATP7B are involved in cisplatin transportation, as previously stated, they are also involved in copper uptake, distribution, and efflux in cancer [73]. According to some proteomic studies, high expression of ATP7A and ATOX1 is associated with poor survival [76,109]. However, the higher expression of ATOX1 with CTR1 to deliver copper showed reduced cisplatin chemoresistance [11]. As a result, the ligand functions as a regulator factor in copper uptake mechanisms, lowering cisplatin chemoresistance [110].

Zinc-chelating structures have a versatile chemistry of donor atoms with different coordination numbers and geometries [99]. The donor atoms are C, N, O, S, or P that form tetrahedron, pentahedron, and hexahedron geometries in cysteine, glutamate, aspartate, and histidine [67,111]. If the donor atom is a water donor molecule, there are tetrahedral, pyramidal, and octahedral coordination geometries [112]. According to Zn’s hard acid nature, the donor atoms O or N are coordinated in the first row rather than S or P in the second row [113]. For instance, the N-donor atom is the primary category with homoleptic and heteroleptic ligands [114]. Due to these varieties, it accesses various arrangements, such as a great assortment of frameworks, from monodentate to hexadentate chelates [115]. As a result, it forms ligands with multiple zinc clusters containing two to four ions in the metal intra-sphere-binding geometry [116] and frequently forms dimeric or polymeric species [61]. Their stereochemistry dominates, with octahedrons in solutions, tetrahedrons in proteins, and a few distorted trigonal bipyramidon examples [117]. Due to its unique chemical features for promoting ligand exchanges, it coordinates into different geometries, resulting in the ubiquitous presence of thousands of proteins and enzymes [118]. This is because of its ability to undergo Lewis activation and nucleophile formation [119]. This catalyst makes it possible to use hydrolytic reactions for DNA cleavage in designing anti-tumour drug activities [120].

### 4.2. Copper and Zinc Ions in Metal–Organic Framework Structures

The copper and zinc ions in MOF interact with organic ligands while remaining physiologically active [121,122]. Despite hydrogen and other bondings, van der Waal and π-π electrostatic interactions load anti-cancer agents or drugs [123]. Copper and zinc ions are endogenously non-toxic transition-metal cations [124]. The common organic ligands are benzene 1,3,5-tricarboxylate (BTC) and tetrakis (4-carboxyphenyl) porphyrin (TCPP) for copper and zeolitic imidazolate (ZIF) for zinc [112]. ZIF may have different formations of ZIF-74 and ZIF-8 and coatings of alginate (Alg) and hyaluronic acid (HA) for different drugs such as ibuprofen [125,126], metformin [127,128], and tetracycline [127,128]. The appropriate combination of them contributed higher efficiencies, such as 80 wt% ibuprofen and 83.5% metformin loadings and a 98% tetracycline clearance rate as shown in Table 1. Cu_3_-(BTC)_2_ and Cu-TCPP may include iron oxide nanoparticles (IONP) and grapheme oxide (GO) for magnetic and photo biosensors, respectively, to trigger doxorubicin release [129,130]. The electrical biosensor combined with either the magnetic or photo biosensor contributed higher efficiencies, such as 40.5 wt % or 45.7 wt % adsorptions and 85.5% or 98.9% release, respectively, as shown in Table 1. As a result, copper and zinc in MOF loading with drugs have different drug performances [91]. This is due to differences in MOF porosities in the physiological setting during host–guest types of interactions [131].

Despite CuZn being an electrical biosensor, it uses an endogenous enzymatic biosensor to stimulate drug release [36]. Thus, both biosensors have been identified as an alternative use of MBD compared to cisplatin [134]. As a result, CuZn in MOF directly integrates drugs to ease production without side effects while remaining biocompatible [91].

## 5. Copper, Zinc, and CuZn in Organic Solvent Formation Structures

**Figure 2:**
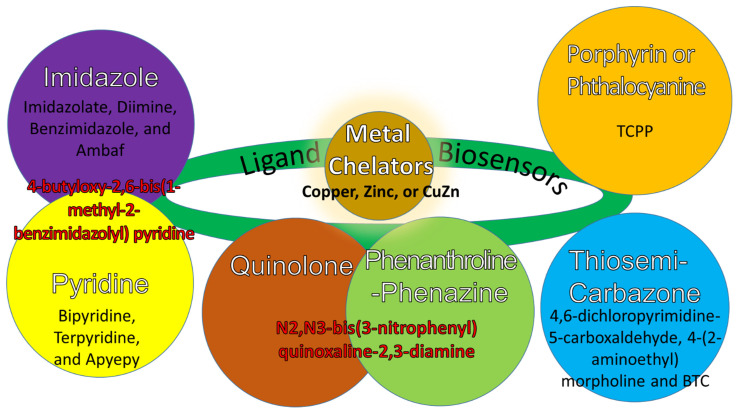
Ligand biosensors link organic solvent groups and metal chelators, resulting in their derivatives and combined derivatives.

Ligand biosensors link organic solvents and metal chelators such as copper, zinc, and CuZn [135]. The organic solvents are mainly classified into imidazole, pyridine, quinolone [136], phenanthroline–phenazine [102], thiosemicarbazone [2,137], and porphyrin or phthalocyanine [138]. The relationship between their formation structures is their derivatives, as described in Figure 2. For instance, the imidazole and pyridine groups [139] have their derivatives of imidazolate, diimine, benzimidazole, and Ambaf; and bipyridine, terpyridine, and Apyepy, respectively [112]. Both groups are combined to form a derivative of 4-butyloxy-2,6-bis(1-methyl-2-benzimidazolyl) pyridine. Furthermore, both quinolone and phenanthroline–phenazine groups are combined to form a derivative of N2,N3-bis(3-nitrophenyl)quinoxaline-2,3-diamine. For the thiosemicarbazone group, their derivatives are 4,6-dichloropyrimidine-5-carboxaldehyde, 4-(2-aminoethyl)morpholine, and BTC. Lastly, the porphyrin or phthalocyanine group has photoactivable properties with the TCPP derivative.

### 5.1. CuZn in Planar Aromatic Structures

The planar aromatic structures with 2,20-bipyridine, quinoline, and 1,10-phenanthroline are popular choices for medicinal chemists [140]. This discovery demonstrated the ability of CuZn to be linked together using phenanthroline ligands to form an NSAID [16,141]. For instance, NSAIDs such as naproxen, ibuprofen, and mefenamic acid have exhibited synergistic anti-proliferative and anti-cancer effects [142]. In particular, zinc with DNA ligands has always demonstrated remarkable anti-inflammatory properties [143]. For instance, zinc(II) compounded with a 1,10-phenanthroline-5,6-dione ligand had similar anti-tumour activity to copper(II) compounded with a phenanthroline–phenazine ligand, as stated previously. Furthermore, CuZn interacts with DNA in the phenanthroline ligand via bidentate chelates in aromatic rings, resulting in anti-proliferative activities [144]. For instance, zinc compounds showed cytotoxic activity and lower IC_50_ values that indicated the cyclooxygenase pathway was inhibited for anti-inflammatory activity [145]. The cytotoxic activities of zinc compounds also showed better resistance than cisplatin.

These CuZn structures intercalate DNA without causing intrinsic toxicity compared to diimines [67]. Moreover, imidazolyl derivatives are the most commonly used N-donor ligands conjugated to active moieties [18]. This is because of their different hapticities and excellent coordination abilities, which are mainly accessible through phenyl ring substitution [146]. The benzimidazole derivatives are their representatives, which consist of 61% (22 of 36 Zn in ZBD) [147]. The Cu and Zn compounds with the benzimidazole–pyridine-quinoline ligand were synthesised and found to have good anti-tumour activity [148]. Furthermore, the anti-tumour activity of tetrahedral copper derivatives (average IC_50_ of 18.91 μM at 72 h) is better than that of zinc derivatives (average IC_50_ of 57.25 μM at 72 h) [149]. Another benzimidazole example, 4-butyloxy-2,6-bis(1-methyl-2-benzimidazolyl) pyridine, was also synthesised with CuZn to form six-coordinated tridentate complexes with distorted octahedral configurations [150]. Their anti-tumour activity findings are that copper(II) derivatives (IC_50_ = 26.09 μM) outperform zinc(II) derivatives (IC_50_ = 46.13 μM), followed by cisplatin (IC_50_ = 43.99 μM) [151]. These copper(II) complexes undergo irreversible redox processes, demonstrating the importance of metal nature in biological activity [148]. Additional Schiff-based for ligands, 4,6-dichloropyrimidine-5-carboxaldehyde and 4-(2-aminoethyl) morpholine, were synthesised with CuZn again, which have the same anti-tumour activity findings that copper(II) outperforms zinc(II), followed by cisplatin [151].

### 5.2. CuZn in Schiff-Based and Schiff-Paired Structures

Schiff-based MBDs are one of the most representative classes of ligands, mainly due to their ease of synthesis and versatility in terms of pharmacological properties [152]. These ligands are tridentate Schiff-based, which gives them high flexibility to coordinate O and N donor atoms [153]. The promising pharmacologically active metal compound is MBD with an N-donor atom and Schiff-based [152]. This is because it has different hapticities to link with CuZn acceptors [59]. The fascinating interests of biosensors are generated as cleavage agents, potential DNA-targeted anti-tumour drugs, and cancer chemotherapeutic agents while conjugating with the DNA gene in catalysis and bio-inorganic systems [54]. Their common pharmacological properties are anticancer, antibacterial, and urease inhibitory activities, resulting in DNA molecule cleavage and DNA duplex cross-linking after interacting with DNA [120]. This MBD has been extensively studied because it has a great impact on cytotoxic activities against various malignant tumours [154].

Either copper or zinc was used to synthesise with either 2-[N-(1H-benzimidazol-2-ylmethyl)ethanimidoyl]-aniline (Ambaf) or 2-(pyridin-2-yl)-N-[1-(pyridin-2-yl)ethylidene]-ethanamine (Apyepy) [155]. Their products are [Cu(Ambaf)H2O]^2+^, [Zn(Ambaf)H2O]^2+^, [Cu(Apyepy)OH]^+^, and [Zn(Apyepy)OH]^+^. They are intercalated with the phosphate groups in DNA [156] to pair electrostatically [157]. In non-tumorigenic P4 fibroblast tests on anti-proliferative activity against human sarcoma cancer cells, the [Zn(Apyepy)OH]^+^ complex with IC_50_ > 140 μM was found to be less cytotoxic than the [Zn(Ambaf)H2O]^2+^ complex with a range of 47 to 71 μM [155]. Furthermore, copper(II) analogous complexes have been discovered to be less cytotoxic than those of zinc(II) complexes [158]. The higher cytotoxicity of the zinc(II) complexes may be due to their photochemical properties [159], as a significant fluorescence increase was observed by interaction with calf thymus DNA [160]. As a result, there is a good correlation between cytotoxicity in anti-proliferative action and cellular metal uptake.

Further investigation revealed that zinc(II) compounds with two benzimidazole-derived pair ligands were synthesised to interact with human serum albumin and DNA, and significant binding propensity was found [161]. Furthermore, their nuclease activities were analysed for pBR322 DNA in order to confirm their potential to cleave DNA [162]. Their IC_50_ values were discovered to be higher than those of PBD and CBD, indicating the lowest cytotoxicity [161]. In another investigation, a CuZn octahedron with different N2,N3-bis(3-nitrophenyl)quinoxaline-2,3-diamine ligands was synthesised to intercalate in DNA [163]. These findings demonstrated that CuZn had more effective DNA cleavage and anticancer activity in HeLa cell lines than free ligands. However, further study is needed to find out whether zinc(II) complexes have lower cytotoxicity than copper(II) complexes.

## 6. Ligand Degradation Properties in Anti-Chemoresistance

**Figure 3:**
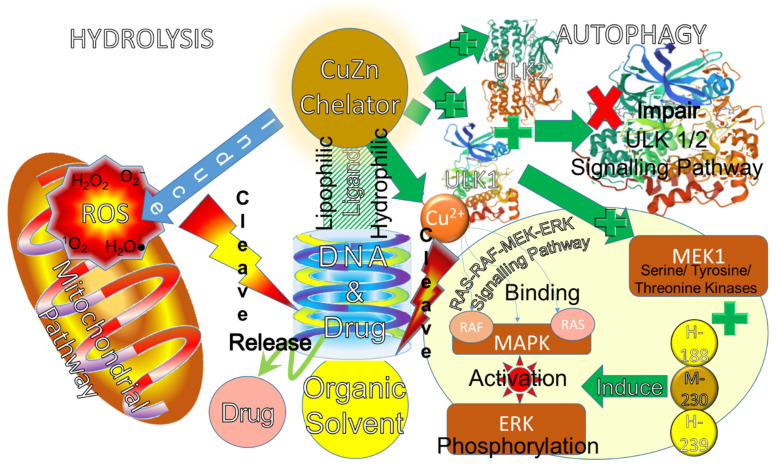
Hydrolysis and autophagy degradation mechanisms of lipophilic or hydrophilic ligand biosensors [40,165,166,167,168]. Reprinted with permission.

Hydrolysis and autophagy are the two main processes of ligand degradation [164] in the copper, zinc, and CuZn complexes. The relationship between the two degradation mechanisms of hydrolysis and autophagy and both lipophilic and hydrophilic ligand biosensors is elaborated in Figure 3. Their induced and cleaved processes for drug release via the mitochondrial and rat sarcoma virus (RAS)-rapidly accelerated fibrosarcoma (RAF)-serine/tyrosine/threonine kinases (MEK)-extracellular signal-regulated kinase (ERK) signalling pathways [40] are highlighted. The copper(II) ions are used to bind with mitogen-activated protein kinases (MAPK) such as RAS and RAF, resulting in no ion for Unc-51-like kinase (ULK)-1/2 bonding.

Hydrolysis is one of the ligand degradation processes used to overcome PBD chemoresistance [169]. For a drug candidate, its stability, solubility, and permeability are determined by the ligand hydrolysis [170]. Their hydrolytic properties are determined by first degrading to either lipophilic or hydrophilic ligands with lipids or water [171]. After MBD is hydrolyzed, the metal compound and drug are released. For instance, NSAID and MBD are developed for anti-cancer activities using conventional approaches such as their organic motifs, frameworks, and donor atom sets [16,141]. Another instance is that copper(II) compounds with either thiosemicarbazone or phenanthroline–phenazine ligand [172,173] exhibit superior anti-tumour activity when compared to metallodrugs or cisplatin [174]. This is mediated by hydrolytic mitochondrial pathways [7] that cleave DNA by oxidatively inducing intrinsic reactive oxygen species (ROS) [175]. Thiosemicarbazone ligands are tridentate [176] structures that comprise many compounds of R1R2 C=N-NH-(C=S)-N R3R4 [55]. This copper(II) in CBD has an active centre in the coordination of Schiff-based ligands [177,178] for a large number of metalloproteins [179]. In order to protect against this oxidative stress, caveolin-1 [180] stabilises ATP7A in vascular tissue to activate superoxide dismutase (SOD)-3 delivery for endothelial function [181]. Furthermore, copper(II) compounded with carbazone inhibited S-phase cell cycle proliferation, which led to cyclin or CDK suppressions and lower IC_50_ values [182]. As a result, the characteristic of this ligand class in copper complexes is its low solubility in water [183]. Their IC_50_ values, which range between 2 and 80 μmol.L^−1^, are vital for drug design to circumvent cisplatin resistance [184]. According to the findings, these complexes showed a lower range of IC_50_, from 0.001 to 0.5 μmol.L^−1^ in HeLa cells, compared to cisplatin’s 18 μmol.L^−1^ [185,186]. This resulted in a greater spread of damage action on all organelles as well as apoptotic death signalling [187].

Another process of ligand degradation is autophagy [188], which copper and zinc complexes use to overcome PBD chemoresistance [189]. For instance, copper binding with ULK1 [165,166] and ULK2 [167] can modulate autophagy activities. The copper-induced mutation of the binding motif ULK-1 and ULK2 (ULK1/2) [168] impairs the ULK1/2-dependent signalling pathway [190]. The amino acid sequence of ULK1/2 is similar to that of MEK-1 [100], which comprises high-affinity copper(II) binding with histidine (H)-188, methionine (M)-230, and H-239 [191]. MEK1 induces ERK phosphorylation [192] that will activate MAPK in tumours such as RAS and RAF. As a result, copper binding activates MAPK to communicate in the RAS-RAF-MEK-ERK signalling pathway [191]. These protein kinases with copper-binding activity induce cell proliferation [193]. However, these copper-binding activities will decrease copper availability, resulting in copper deficiency [25]. Conversely, the increase in copper availability will enhance ULK1/2 activities and autophagy functions [190]. Thus, copper modulates autophagy functions [194] in tumour-associated macrophages and bone marrow myeloid precursor recruitment [195] that promote changes in the tumour microenvironment to reduce tumours indirectly.

## 7. Ligands’ Functions as Biosensors for Osteosarcoma Therapy

### 7.1. CuZn Ligands for Redox Biosensor Functions

The copper(I) in CBD modulates its redox potency through its imidazole-like imine ligands to treat cancer [196]. Moreover, zinc is a redox-inert substance in biology and an antioxidant used in cancer treatments [197]. During the catalysis of endogenous substrates, CuZn releases ions and generates ROS [198]. This ROS triggers oxidative stress by attacking the Cu-Zn SOD in extracellular form [199]. As a result, without taking zinc’s pleiotropic functions into account, it is not true that oxidative stress decreases in response to zinc deficiency or a lack of antioxidant mechanisms [197]. This is because zinc complexes interfere with mitochondrial metabolism’s ability to generate ROS and transport it through its special cell incubation medium [200]. This oxidative stress is amplified by CuZn reactivity, resulting in partial or total damage to bilayer lipid membranes, protein alterations, and gene DNA functions [201,202]. For instance, complexes of zinc penta-coordinated with binuclear ligands are more active than complexes of zinc hexa-coordinated with mononuclear ligands [203]. This is due to ROS overproduction triggering DNA damage, resulting in good DNA accumulation and cellular uptake via intrinsic pathway-dependent apoptosis [204]. Thus, the unbound CuZn ions or free radicals eventually interfere with the cell cycle at different levels, resulting in cell disorders, necrosis, and apoptosis [204].

For instance, STEAP converts copper into copper(I) that binds the cytochrome C oxidase (Cox) copper chaperone [19]. This results in activation of the Cox17 gene for SOD1 delivery [205]. SOD1 is a cytoplasmic protein and also a transcription factor that regulates oxidative stress in the nucleus [206]. ATOX1 is a metallochaperone protein and a protective agent against oxidative stress that binds copper to indirectly modulate cell proliferation and nucleus migration [207]. In the trans-Golgi network, ATPase7A and ATPase7B proteins donate copper ions to ATOX1 to secrete cuproenzymes such as lysyl oxidase (LOX) and ceruloplasmin [208]. In cancer cell lines, LOX activity is inhibited by silencing the ATP7A gene, which reduces tumour growth and metastatic potential [209]. However, the loss of function of ATP7A in cell proliferation showed toxicity due to copper excess [39]. Another instance of converting copper(I) to copper(II) by STEAP4, which is a metalloreductase, induces the inflammatory cytokine interleukin (IL)-17 for CTR1 transportation [106]. This will increase copper uptake and activate the cytoplasmic X-linked inhibitor of apoptosis protein (XIAP) [210]. XIAP suppresses caspase-3 function with a ubiquitin E3 ligase activity that impairs apoptosis, thus allowing cell proliferation [106]. As a result, copper also regulates the activities of cancer cell proliferation and apoptosis.

### 7.2. CuZn Ligands for Photo-Biosensor Functions

The photoluminescence and photosensitive properties are demonstrated by the CuZn ligands such as terpyridine [211], BTC [212], TCPP, porphyrins, and phthalocyanines [146]. This is because of photoactivable N-donor ligands in pyridine-based and porphyrin-Schiff-based systems [211]. Both ligand systems with CuZn intercalate into DNA compounds and wall interactions, thus improving photocytotoxic activity against microorganisms [213]. For instance, zinc–phthalocyanine complexes used in photodynamic therapy (PDT) demonstrated photo-activable N-donor ligands, low dark cytotoxicity, and tumour cell inhibitory effects [214]. This is a good photochemical stability product without photoreaction toxicity, as evidenced by its extremely high IC_50_ values [215].

## 8. Conclusions

Copper and zinc ions are used as metal chelators to bind with an O, N, S, or P donor atom in MOF, planar aromatic, Schiff-based, and Schiff-paired structures. The popular planar aromatic structures are diimine, phenanthroline–phenazine, terpyridine, BTC, TCPP, and phthalocyanine. The metals bond with aromatic rings by using either bi-, tri-, tetra-, penta-, or hexadentate ligands. If the structure is Schiff-paired, they can be extended to octadentate ligands. Schiff-based and MOF structures are easily bonded with CuZn acceptors in different coordination numbers and geometries. Their ligands intercalate with the DNA phosphate groups using hydrogen and other bonding, van der Waal, π-π, and electrostatic interactions. CBD and ZBD are the most promising pharmacological NSAIDs with active metal chelation. This enables zinc to bind excess copper, which avoids genetic disorders and releases oncogenic enzymes, such as ATP7A, ATP7B, CTR1, and ATOX1, to regulate homeostasis. These changes restore the balancing and controlling mechanisms in cellular trafficking during cancer invasion. Therefore, copper(II) had better anti-tumour activity findings than zinc(II), followed by cisplatin. As a result of overcoming cisplatin chemoresistance and having additional low toxicity and fewer side effects, which has emerged as the primary OST strategy in tumoral pathologies.

Both copper and zinc can be regulated by ligand degradation processes such as hydrolysis and autophagy to release their compounds. Both thiosemicarbazone and phenanthroline–phenazine ligands exhibit superior anti-tumour activity when compared to metallodrugs or cisplatin. This is because DNA is hydrolytically cleaved by oxidatively induced intrinsic ROS via mitochondrial pathways. For instance, imidazole-like imine organic solvents are commonly used in conjunction with this redox function. While ROS attacks SOD, redox functions are generated, and oxidative stress is amplified by copper and zinc free radicals’ reactivity. This stress damages bilayer lipid membranes and DNA, causing DNA accumulation and cellular uptake and resulting in cell disorders, necrosis, and apoptosis. For instance, copper exists in two coordination redox states, such as copper(I) and copper(II), which are converted by STEAP and STEAP4, respectively, for CTR1 transportation. Copper(I) is activated by the Cox17 gene to donate ions to ATOX1 via the ATPase7A and ATPase7B proteins, resulting in ATOX1 secreting LOX for SOD1 delivery. Thus, LOX activities are silenced by the ATP7A gene to inhibit tumour growth and metastatic potential in cancer cell lines. The inflammatory cytokine IL-17 activates copper(II), followed by the cytoplasmic XIAP, by increasing intracellular copper uptake. XIAP impairs apoptosis and allows cell proliferation via suppressing ubiquitin E3 ligase activity in caspase-3 function. For instance, autophagy is modulated by copper binding, which activates MAPK to impair the ULK1/2-dependent RAS-RAF-MEK-ERK signalling pathway and induce cell proliferation. As a result, the CTR1 transporter and STEAP4 reductase mechanisms can regulate the copper levels in cancer cells. Nonetheless, CuZn has significant chemotherapeutic potential, especially as biosensors in drug delivery systems. These compounds bonded with terpyridine, BTC, TCPP, and phthalocyanine organic solvents with photo-activable N-donor ligands that demonstrated photoluminescence and photosensitive properties, low dark cytotoxicity, and inhibitory tumour cell effects.

## 9. Challenges and Future

CuZn demonstrated more cytotoxicity against tumour cells than normal cells in chemodynamic therapy (CDT) [4,216]. They are commonly used to endogenously catalyse hydrogen peroxide (H_2_O_2_) into hydroxyl radicals (•OH) by Fenton-like reactions [107,217]. This •OH generation of redox reacts with copper(I) to release zinc protoporphyrin IX, which strongly inhibits the activity of the typical enzymatic antioxidant heme oxygenase-1 [218]. As a result, ROS generation inhibits tumour growth and causes serious oxidative damage to cellular constituents, resulting in cell death without adverse side effects [68]. However, SOD1 was found to respond differently to two proteins, tyrosine 3-monooxygenase/tryptophan 5-monooxygenase activation protein (YWHA)-zeta and YWHA-epsilon, depending on its redox status in terms of structural dependences, protein degradation, and metabolic implications, [219]. This is a new, unorthodox role of SOD1 as a major redox enzyme in scavenging superoxide radicals (O_2_^−^) that creates different perspectives of insight diagnosis to map protein binding domains in co-crystalline structures [42,204]. Further research should be conducted to characterise molecular mechanisms and their metabolic relevance in physiological conditions [30].

PDT is a light-required therapy [220] that produces oxygen and ROS to reduce antioxidant enzymes such as catalase and SOD [221]. An antimicrobial PDT trial using CuZn compounds synthesised with SOD found the highest bacterial concentrations with 1.2 μg/mL reductions in 30 min. of inhibition time [222]. These findings in two mediums, diethydithiocarbamate and methylene blue, indicated a new possibility for an antimicrobial PDT study [223]. Since OST is a long-term treatment, more research into its microbial and bacteria-curing mechanisms is needed. Another PDT and CDT consist of dual-activated Zn-TCPP and Cu-diethyldithiocarbamate (DTC)_2_ biosensors, respectively, which have antitumor activity and prevent systemic toxicity [91]. ROS are stimulated to cleave the hyaluronic acid-conjugated Cu(DTC)_2_ prodrug by photo-trigger reactions on Zn-TCPP [224]. This will release DTC and Cu to re-induce ROS [225]. This method avoids administering Cu-(DTC)_2_ directly, which causes severe systemic toxicity [226]. In contrast, insufficient endogenous copper can severely limit the antitumor activity of Cu(DTC)_2_ and disulfiram generation.

Both drug carriers, gelatin/chitosan/hydroxyapatite [227] and folate-decorated Alg/polydopamine/paclitaxel (FA-Alg/PDA/Ptx) [228], used CuZn as biosensors in targeted therapy that demonstrated pH sensitivity and precise delivery of antitumor efficacy [229]. This FA-Alg/PDA/Ptx drug carrier had good encapsulation, loading, and IC_50_ efficiencies of 75.2 ± 1.54%, 18.54 ± 2.31%, and 150 ± 2.58 μg/mL, respectively, indicating remarkable efficiency and drug potency [228]. Despite having a ȥ-potential of −31.4 ± 1.54 mV [228], the electrical biosensor potency has not been studied. As a result, PDT, CDT, and pH are being studied for mitochondrial membrane targeted therapy in cancer, with less off-target toxicity and more desirable therapeutic effects [230]. Nonetheless, the challenges of CuZn in overcoming MBD and PBD chemoresistance should be investigated further because a contrary study found that zinc(II) complexes have lower cytotoxicity than copper(II) complexes [163]. This is a critical caution because the amount of copper in our bodies is critical and must be carefully regulated.

## Figures and Tables

**Table 1:** Copper and zinc in metal–organic framework loading with drugs and their performances.

Drug Carrier	Drug	Efficiency	Refs.
ZIF-74	Ibuprofen	80 wt% loading efficiency	[125,126]
ZIF-8/Alg	Metformin	83.5% loading efficiency, and 6.68 wt.% payload.	[132,133]
ZIF-8/HA	Tetracycline	98% clearance rate under acidic conditions and pH-responsive.	[127,128]
Cu_3_-(BTC)_2_/IONP	Doxorubicin	Adsorbed 40.5% and released 85.5% at pH 5	[129,130]
Cu-TCPP/GO		Adsorbed 45.7 wt.% and released 98.9% at pH 5.	

## Data Availability

Not applicable.

## Abbreviations

MBD: metal-based drugs; OS, osteosarcoma; CuZn, copper and zinc; PBD, platinum-based drugs; CBD, copper-based drugs; ZBD, zinc-based drugs; NSAID, non-steroidal anti-inflammatory drugs; OST, OS therapy; MOF, metal–organic frameworks; CDK, cyclin-dependent kinase; ATP, ATPase; CTR, copper uptake protein; ATOX1, antioxidant protein 1; STEAP, 6-transmembrane epithelial antigen of prostate reductase; BTC, benzene 1,3,5-tricarboxylate; TCPP, tetrakis (4-carboxyphenyl) porphyrin; ZIF, zeolitic imidazolate; Alg, alginate; HA hyaluronic acid; IONP, iron oxide nanoparticles; GO, grapheme oxide; Ambaf, 2-[N-(1H-benzimidazol-2-ylmethyl)ethanimidoyl]-aniline; Apyepy, 2-(pyridin-2-yl)-N-[1-(pyridin-2-yl)ethylidene]-ethanamine; RAS, rat sarcoma virus; RAF, rapidly accelerated fibrosarcoma; MEK, serine/tyrosine/threonine kinases; ERK, extracellular signal-regulated kinase; MAPK, mitogen-activated protein kinases; ULK, Unc-51-like kinase; ROS, reactive oxygen species; SOD, superoxide dismutase; H, histidine; M, methionine; Cox, C oxidase; LOX, lysyl oxidase; IL, interleukin; XIAP, X-linked inhibitor of apoptosis protein; PDT, photodynamic therapy; CDT, chemodynamic therapy; YWHA, tyrosine 3-monooxygenase/tryptophan 5-monooxygenase activation protein; DTC, diethyldithiocarbamate; FA, folate-decorated; PDA, polydopamine; Ptx, paclitaxel.
